# Dysrhythmias resulting from surgical manipulations of pituitary tumour and hydrogen peroxide irrigation of surgical wound

**DOI:** 10.4103/0019-5049.68397

**Published:** 2010

**Authors:** Hemanshu Prabhakar, Gyaninder Pal Singh, Ashish Bindra, Zulfiqar Ali

**Affiliations:** Department of Neuroanaesthesiology, Neurosciences Center, All India Institute of Medical Sciences, New Delhi - 110 029, India; Department of Anaesthesia and Intensive Care, Government Medical College, Srinagar, Jammu and Kashmir, India

Sir,

Dysrhythmias are well known and not of uncommon occurrence in neurosurgical procedures.[1] The proximity of the surgical site to vital centers and initiation of trigemino-cardiac reflex (TCR) is often implicated. Hydrogen peroxide irrigation is commonly performed to clean the surgical wound and achieve haemostasis. The use of hydrogen peroxide is also not without haemodynamic complications.[2] We report a case of a female undergoing surgery for pituitary tumour who suffered dysrhythmias both during manipulation of the tumour, as a result of TCR and at the time of irrigation of hydrogen peroxide.

A 16-year-old female was admitted to our neurosurgical unit with presenting complaint of acromegalic features. Magnetic resonance image (MRI) of the head revealed a 5.2 × 3.2 × 4.5 cm lesion in the sellar extending to suprasellar region, suggestive of pituitary tumour. The patient was scheduled for elective craniotomy and tumour resection. The patient was premedicated with intramuscular glycopyrrolate 0.2 mg, one hour prior to surgery. General anaesthesia was induced with fentanyl 2 mcg/kg and thiopentone 4-5 mg/kg. Tracheal intubation was facilitated with rocuronium 1 mg/kg. An arterial line was placed in the dorsalis pedis artery of left foot. Anaesthesia was maintained with isoflurane (MAC~ 1 ± 0.2) in a mixture of O_2_ and N_2_O (1:2). At the time of tumour resection, there occurred sudden bradycardia and hypotension. There was decrease in heart rate from 70 beats per minute (bpm) to 52 bpm and blood pressure dropped from 110/76 to 68/45 mmHg. The surgeon was immediately informed. No sooner the surgical stimulus stopped, the heart rate and blood pressure reached the pre-stimulus values. Surgery was allowed to continue. To achieve complete haemostasis, the surgeon irrigated the surgical wound with 10 ml of diluted 3% hydrogen peroxide solution (1:1). The effervescent solution resulted in a sudden decrease in heart rate from 76 bpm to 45 bpm. The solution was immediately aspirated and field irrigated with normal saline. The heart rate gradually improved over next 15 seconds. No further use of hydrogen peroxide was allowed. Rest of the surgical and anaesthetic course was uneventful. At the end of 5 hour surgery, anaesthetics were discontinued, neuromuscular block reversed with neostigmine and glycopyrrolate and tracheal extubation done.

TCR is a well recognised phenomenon that classically comprises bradycardia, arterial hypotension, apnea and gastric hypomotility.[3] TCR is a reproducible phenomenon and has been reported during cranio-facial surgery and during surgery with the cerebellopontine angle, petrosal sinus, orbit and trigeminal ganglion.[4] It may be produced during surgery as early as elevation of skin flap for craniotomy.[5] Stimulation of any division of the trigeminal nerve can produce this reflex. Pituitary gland is flanked by the cavernous sinuses and the maxillary and ophthalmic divisions of the trigeminal nerve are situated on the lateral wall of cavernous sinus. Stimulation of these divisions could have triggered the reflex. The second remarkable event in our patient was sudden bradycardia having a temporal relationship with hydrogen peroxide irrigation. This could have possibly been due to stimulation of the hypothalamus which is in close proximity to the pituitary gland. Parasympathetic outputs are organised in the anterior hypothalamus, sympathetic pathways in the posterior hypothalamus. It is likely that hydrogen peroxide irrigation may have generated an intense parasympathetic activity leading to bradycardia. The liberated oxygen may have mechanically stimulated hypothalamus. Since hydrogen peroxide produces an exothermic reaction on contact with organic tissues, the possibility of raised temperature in the area of hypothalamus cannot be ruled out. This raised temperature may have been responsible for the dysrhythmia.[3]

We suggest that the possibility of TCR should be kept in mind during surgery for pituitary tumours.
